# Reasons for compliance or noncompliance with advice to test for hepatitis C via an internet-mediated blood screening service: a qualitative study

**DOI:** 10.1186/1471-2458-11-293

**Published:** 2011-05-10

**Authors:** Freke R Zuure, Titia Heijman, Anouk T Urbanus, Maria Prins, Gerjo Kok, Udi Davidovich

**Affiliations:** 1 Cluster Infectious Diseases, Department of Research, Public Health Service of Amsterdam, Amsterdam, the Netherlands; 2 Center for Infection and Immunology Amsterdam (CINIMA), Academic Medical Center (University of Amsterdam), Amsterdam, the Netherlands; 3 Department of Work and Social Psychology, Faculty of Psychology and Neuroscience, Maastricht University, Maastricht, the Netherlands

## Abstract

**Background:**

Hepatitis C virus (HCV) is mainly transmitted by exposure to infected blood, and can lead to liver cirrhosis and liver cancer. Since the onset of HCV and the development of liver cirrhosis usually are asymptomatic, many HCV-infected individuals are still undiagnosed. To identify individuals infected with HCV in the general population, a low threshold, internet-mediated blood testing service was set up. We performed a qualitative study examining reasons for compliance and noncompliance with advice to test for HCV via the online blood testing service.

**Methods:**

Semistructured telephone interviews were conducted with 33 website visitors who had been advised to test for HCV (18 testers, 15 non-testers). Transcribed interviews were analyzed qualitatively and interpreted using psychosocial theories of health behavior.

**Results:**

Reasons for testing pertaining to the online service were: the testing procedure is autonomous, personalized test advice is provided online, reminder emails are sent, and there is an online planning tool. Reasons for testing not specific to the online service were: knowing one's status can prevent liver disease and further transmission of HCV, HCV is curable, testing can provide reassurance, physical complaints are present, and there is liver disease in one's social environment. Service-related reasons for not testing pertained to inconvenient testing facilities, a lack of commitment due to the low threshold character of the service, computer/printing problems, and incorrectly interpreting an online planning tool. The reasons for not testing that are not specific to the online service were: the belief that personal risk is low, the absence of symptoms, low perceived urgency for testing and treatment, fear of the consequences of a positive test result, avoiding threatening information, and a discouraging social environment.

**Conclusions:**

Features specific to the online service played a significant role in motivation to test for HCV above and beyond the more conventional perceived health benefits of HCV testing. However, some online specific features were considered problematic and need to be adapted. Methods and strategies for dealing with these impeding factors and for improving compliance with testing via the online service are outlined.

## Background

Hepatitis C virus (HCV) infection, caused by a blood-borne virus and first identified in 1989, is a major public health problem. Worldwide an estimated 123 million individuals are HCV antibody positive, [1] approximately 75% of whom are chronically infected and at risk for the development of cirrhosis, liver cancer, and death [2,3]. In chronically infected patients, the onset of HCV itself and the development of cirrhosis are usually asymptomatic [2,4]. Therefore, many infections remain undetected or are diagnosed late. On the basis of mathematical modeling, the HCV-related morbidity and mortality rates in high-income countries are expected to at least double in the next 2 decades [5,6]. Because successful combination therapy for HCV became widely available in 2001 [7-11] and an era of new therapeutic options is expected shortly [12,13], the challenge now is to identify as many HCV-infected individuals as possible. Consequently, the Public Health Services of Amsterdam and South Limburg introduced the Hepatitis C Internet Project, a pilot study aimed at identifying undiagnosed HCV-infected individuals in the general population.

In the Netherlands, the estimated HCV prevalence is low (0.1-0.4% [14]). Therefore, the strategy used in the Hepatitis C Internet Project consisted of a public media campaign that addressed HCV risk factors and referred risk groups to an online HCV risk assessment questionnaire at http://www.heptest.nl[15]. Individuals who visited the website and were identified by the questionnaire as at risk were advised to get tested for HCV and were immediately offered the opportunity to arrange, online, a free and anonymous HCV blood test. The website also provided information about HCV risks that was tailored to the individual's risk profile and emphasized the severity of HCV infection, its often asymptomatic onset, and the benefits of treatment. It also explained the testing procedures, stating that the blood test procedure included an initial HCV antibody test, a follow-up test for those who tested positive, and a direct referral to the hospital for those infected with HCV. Individuals could arrange the blood test themselves by printing out a laboratory form that contained a personal identification code with which participants could anonymously obtain their test result online seven days after testing. The form also included addresses and opening hours of the participating low threshold test locations. In order to increase the test uptake, individuals were offered an online planning tool for testing where they could specify the date, time, and location upon which they would have their blood drawn for the HCV test. The tool explicitly mentioned that it did not result in an actual appointment with the laboratory and that individuals later could decide to take their test at a different date, time, or location. The tool was considered advantageous because, according to the theory of Implementation Intentions [16], detailed planning of when and how to execute an intended action facilitates the actual performance of the behavior. In addition, individuals could subscribe to an email and/or a mobile phone Short Message Service (SMS) reminder system if they wanted to receive a reminder message for blood testing five days after they completed the risk assessment questionnaire.

While 28% (*n *= 420) of the individuals who completed the risk assessment questionnaire and were found to be at risk for HCV infection (*n *= 1,480) complied with the test advice and were tested for HCV, a substantial proportion (72%) failed to visit the test locations. Because the online testing service is new, it is unclear which service-related factors promoted or impeded website visitors' decision to test for HCV. Understanding why some complied with the advice to test through the online service and others did not is vital to not only the further implementation of this service but also to the improvement of HCV testing campaigns in general. Therefore, this study investigated reasons for compliance and noncompliance with the HCV test advice obtained through the online risk screening tool and focused particularly on the role of the online blood testing procedures in that process. A descriptive qualitative design was used to be able to explore and understand the participants' views and motives with regard to HCV testing.

### Theoretical background

The health belief model [17,18], the theory of planned behavior [19], and the extended parallel process model [20] were used as theoretical bases for the interpretation of the findings. The health belief model focuses on perceived severity of and vulnerability to a disease (perceived threat), perceived barriers to and benefits of executing the behavior (expectations regarding the outcomes of the positive health behavior), perceived self efficacy (the degree to which one perceives oneself capable of executing the health behavior), and cues to action (stimuli which trigger the cognitive processes that lead to the health behavior). Applied to the context of HCV screening, the likelihood of HCV testing increases when perceived threat of HCV is high, perceived barriers of testing are low, perceived benefits of testing are high, self efficacy for testing is high, and relevant cues for action are present.

The theory of planned behavior suggests that behavior is determined by more than just health beliefs. According to the theory of planned behavior, attitudes (personal evaluations of the behavior based on behavioral beliefs), subjective norms (perceptions of other people's evaluations of the behavior based on normative beliefs), and behavioral control (perceived control over the execution of the behavior based on control beliefs; similar to self-efficacy) determine the intention to engage in a behavior. Behavioral intention is presumed to best predict behavior. However, actual behavioral control (e.g. lack of control due to environmental factors) can also directly influence behavior. Applied to the context of HCV screening, the likelihood of HCV testing increases when attitudes towards testing are positive, when subjective norms favor HCV testing, and when perceived behavioral control is high.

The extended parallel process model also focuses on health beliefs but is more specific than the health belief model with regard to the role of emotion in responses to a perceived health threat. According to the extended parallel process model, health threats can cause individuals to engage in either danger control or fear control processes. Danger control is aimed at reducing the health threat through cognitively processed adaptive responses (e.g. seeking testing and treatment), whereas fear control is aimed at reducing unpleasant feeling related to the health threat. Fear control often results in maladaptive responses such as message avoidance and defensive reactions (e.g. denial of risk). Whether individuals engage in danger or fear control processes depends on the degree to which threat, self efficacy, and response efficacy (the extent to which the recommended behavior is expected to effectively reduce the threat) are perceived to be present [20]. Medium to high perceived threat combined with high perceived efficacy will most likely result in danger control responses while high perceived threat combined with low perceived efficacy will most likely lead to fear control responses. In a study conducted with men who have sex with men (MSM) by Mikolajczak et al. [21], men at risk for HIV mentioned both the fear of testing HIV-positive and a low perceived risk of HIV infection as reasons for not testing, thus implying that cognitive dissonance reduction takes place. Applied to the context of HCV screening, the extended parallel process model would suggest that the likelihood of HCV testing is greatest when individuals perceive the threat of HCV as moderate to high and possess high levels of perceived self efficacy and response efficacy.

## Methods

### Recruitment and Sample

Because the Hepatitis C Internet Project was anonymous, only individuals who had subscribed to the reminder service could be contacted for participation in this study. To note, those who subscribed to the service were informed that they could receive an email invitation for participation in a study. Recruitment took place among these individuals between May and July 2007 and between May and July 2008 (*n *= 97). The invitation sent by email briefly explained the study procedures and indicated that the aim of the study was to improve the project by hearing the opinions of participants. The invitation was sent at least three weeks after the potential participant's website visit, in order to provide the participant with sufficient time to be tested, but no later than three months, in order to reduce potential recall bias. If individuals did not reply to the email invitation within two weeks, an email reminder was sent. Recruitment of participants continued until data saturation [22] was reached, i.e. until no new reasons for compliance or noncompliance emerged from three consecutive interviews. In total, 33 interviews were conducted. Information regarding demographics (sex, level of education, and country of birth) and HCV risk factors were obtained from the online risk assessment questionnaire data. Age was asked during the interview.

### Procedure

Semistructured interviews were chosen as they allow flexibility, facilitate empathy, enable the interview to explore new topics, and tend to produce rich data [23]. Interviews were conducted in Dutch by telephone. Telephone interviews were considered the most ideal choice as they lower possible barriers to participation (e.g. travelling to the Public Health Service) and enhance anonymity. Two female researchers conducted interviews of approximately 15 minutes each. Every interview commenced by explaining the purpose of the interview followed by an oral informed consent. The following topics were then addressed: motives for visiting http://www.heptest.nl and filling out the risk assessment questionnaire; feelings about the outcome of the risk assessment; personal perception of risk for HCV infection (this topic was added after the first two interviews); and the reasons for compliance or noncompliance with the advice to test for HCV. The central topic of the interview concerned why participants used or did not use the project's testing service. Follow-up probes (e.g., "Could you explain this further?") were applied to motivate participants to provide a detailed rationale for their test decision. All interviews were audio-taped and transcribed verbatim (quotes are translated). Participants were provided with a gift certificate as reward for their participation.

### Analyses

The transcribed interviews were entered into a database for coding and content analysis using qualitative data analysis software (MAXqda 2007). The data analysis team consisted of four researchers from different disciplines (communication science, biomedical science, psychology, and anthropology). The data analysis consisted of two phases; in the first phase the data were analysed in an inductive manner, not informed by the theoretical frameworks. In the second phase, the results of the first phase were interpreted using the theoretical frameworks. A detailed description of the two phases follows below.

Phase 1: After conducting the first two interviews, two researchers independently coded those interviews. In order to stay as close as possible to the phenomenon described by the participants, coding was inductive and open, not yet classified or interpreted through the theoretical frameworks, and an unrestricted number of facets were expressed in preliminary code names. A discussion meeting with the data analysis team then took place to ensure that all relevant content was incorporated in codes. Furthermore, based on the first two interviews, the team discussed whether additions were needed to the initial interview schedule. Thereafter, two additional interviews were conducted and discussed. This iterative process of interviewing alternated with open coding and a team discussion comprised three rounds. Thereafter, when all 33 transcripts were coded, the team reached consensus on the final code names. The codes had to be concise and self-explaining. If multiple codes were found that refer to a similar principle (e.g., the codes: 'inconvenient opening hours of the laboratories', 'no evening opening hours', and 'needing an appointment for a specific time to be tested at the closest laboratory'), codes were merged together (in this example into 'inconvenient test facilities'), reducing the number of codes. Consequently, the first author then reread the coded segments of the 33 transcripts to confirm that all coded segments fitted in the final code names.

Phase 2: Focusing on the research question, the team then grouped the relevant codes into categories based on the theoretical background of the health belief model, the theory of planned behavior, and the extended parallel process model. Each category was based on at least one code.

### Ethical framework

Prior to the interviews, participants were informed about the purpose of the interview and the fact that they could withdraw from participation whenever they wished (also after finishing the interview), by emailing the researcher that had contacted them via email previously (none of the participants withdrew). Oral informed consent for audio taping the interview was requested before the interview started. To maximize confidentiality, potential personal identifiers were deleted from the transcripts, and only the involved researchers had access to the interview transcripts. The study was approved by the Ethical Committee Psychology of the School of Psychology and Neuroscience, Maastricht University.

## Results

### Sample characteristics

Most participants (91%; 30/33) were born in the Netherlands and female (79%; 26/33). Median age at the time of the interview was 49 years (IQR = 41-62 years). Educational level varied from low (22%) to moderate (19%) to high (59%). Of the 33 participants, 18 (55%) had complied with the test advice and had used the project's testing service. One of these 18 had tested positive for HCV. The participants belonged to various HCV risk groups. The most frequently reported risk was having had a blood transfusion before 1992 (*n *= 16), followed by having the skin pierced in countries with medium to high HCV prevalence (*n *= 13). Other reported risks were former injecting drug use (*n *= 2), frequent use of non-injection illicit drugs (i.e., cocaine, heroine, amphetamine, LSD, GHB and/or poppers; *n *= 1) and living together and sharing bathroom attributes with HCV positive individuals or drug users (*n *= 4). Three participants had multiple HCV risks.

### Reasons for testing related to the online testing procedures

**Figure 1:**
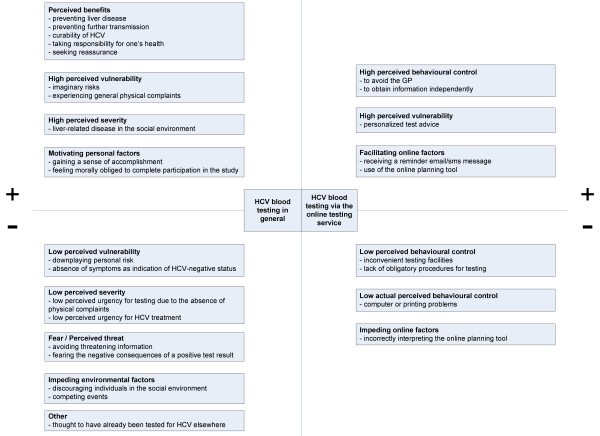
**Reasons for compliance (+) and noncompliance (-) with online advice to test for HCV**. The left section of the figure presents the reasons related to HCV testing in general whereas the right section presents reasons specific to testing via the online blood testing service. *Note*. HCV = hepatitis C virus

From the interviews with participants that had been compliant with the advice to test (N = 18), we identified five reasons for testing that related directly to the online testing procedure (see upper right section of Figure 1). The first reason was that the online testing service allowed individuals access to a test without having to discuss or explain their desire to be tested for HCV with their general practitioner (GP). This reason was labeled '*to avoid the GP*' and is illustrated by the following quotes:

"At that time [years ago], I thought about testing but I didn't do it. [...] The reason is that, back then, you had to visit the GP - it was the standard procedure - and you'd have to tell him or her why you want a test [...] and, with this offer, you can remain anonymous but still get tested." (tester [T]-8)

"I have a lot of health problems. Visits to the GP are time-consuming and, above all, you don't want to be thought of as a whiner. [...] Everytime you have something, you kind of start to dislike to yourself and, by bringing it up with the GP, it's like you are again make a big deal out of things." (T-9)

The second reason for compliance pertaining to the testing service was that the online service enabled users to become well-informed about HCV and the testing procedures without time pressure and at their own pace. The fact that participants '*could obtain information independently*' enabled them to deliberate about whether or not they should test for HCV:

"Well, after doing the risk test and being told that I need to test, then you can search for information yourself and find out what it all means, you know? Then you are not blindly having your blood drawn while you actually know nothing. You can immediately search on the internet. You can look up why or how and what...Then I think, 'It's not so scary, I'll do it'" (T-1).

The third reason was that there was '*personalized test advice*'. The tailored feedback on risk factors provided motivation to test, as illustrated by the following quote:

"Well it [the personal advice] is so clear that you feel compelled to follow the advice you receive" (T-8).

The fourth reason was that participants had been reminded to get tested. '*Receiving a reminder e-mail/sms message*' alerted individuals to the test advice and promoted testing, as illustrated by the following the quote:

"Well, actually, I think if I didn't get that reminder of yours, it would have ended up in the back of my mind, like something I would have to do some time. [...] Without the reminder, I probably wouldn't have gotten tested." (T-12)

The final reason for compliance related to the online service was the availability of the online planning tool. The '*use of the online planning tool*' stimulated participants to test:

"I think it's a helpful tool. They ask you what date you want to go. I thought, 'Hey, that's good. I'll just pick that date. I'll do it. I'll just put it in my day-planner and I'll do it'" (T-7).

### Reasons for testing unrelated to the online testing service

We identified ten reasons for testing that were unrelated to the online testing service (see upper left section of Figure 1). First, participants mentioned health gain from early detection of HCV. This was labeled as '*preventing liver disease*' and is illustrated by the following quote:

"These diseases always start small. They are invisible and, later on, they develop further and, at a certain point, you're too late for treatment. You know, it gives you problems. If you find it at an early stage, you may be able to cure or treat it." (T-4)

Secondly, participants reported testing because the undetected virus could spread to other people. This reason was labeled as '*preventing further transmission*' and is illustrated by the following quote:

"I thought, 'Well, for goodness' sake, let me get the test.' [...] also because I could infect others with it" (T-7).

A third reason for testing was labeled '*curability of HCV*'. This reason focused on the fact that there are treatment options for HCV when diagnosed. One participant said,

"I also read that there are medications and stuff available, so I thought ooohkay. [...] I thought, 'Well, this is not very scary, I will do it.' You get me?" (T-1).

Another participant stated, "I heard that if you have it, it can be effectively treated, at least if you've detected it at an early stage. That's why I reacted immediately" (T-6).

Furthermore, some participants expressed that caring for one's own health and body was imperative. '*Taking responsibility for one's health*' was thus one of the reasons to test for HCV:

"Look, when I hear about something like this, I take action immediately. It is my body and I believe that we should care for our bodies. And when you are offered something like this, well, then you should do it" (T-18).

Some participants mentioned that they got tested because they wanted to know their HCV status. They were not scared of the test results but reported that they were '*seeking reassurance*':

"I wasn't afraid that something was wrong but, yes, I wanted to be sure." (T-5); and "I have other things, I mean unpleasant things [medical conditions] so I liked being able to exclude something" (T-9).

Several participants had incorrect perceptions regarding HCV risks. They had experienced certain events that they considered to pose a risk. Although these events posed no actual risk, they did increase perceived risk and motivated individuals to test. We labeled this reason as '*imaginary risks*':

"I've had numerous medical examinations and much more. I've had a stroke, three TIAs. [...] I used to go for walk in wooded areas and I've been bitten by ticks [...] so I thought, "Oh, oh maybe it [being HCV positive] could be because of all that." (T-2)

Also, some participants reported testing because they were '*experiencing general physical complaints*'. One participant said the following:

"It said that you could be carrier for a long time and that it won't manifest itself - only maybe in a much later stage - that it can take years. And yes, well maybe it's because lately I have had a lot of complaints that I never had before. I thought well, 'For goodness' sake, let me get the test.'" (T-7)

In addition, knowing people with liver-related diseases was mentioned as a reason for testing. This was labeled as '*liver-related disease in the social environment*' and is illustrated by the following quote:

"At this time, I have acquaintances who are dying because of their liver. So I think the liver is very important" (T-18).

Another reason for testing was based on the principle of finishing what you started and was labeled as '*gaining a sense of accomplishment*':

"Well, I tested because I think, 'Well, I want to know, finish this, just do it.'" (T-17).

"There was no specific, no special reason, just to have it done" (T-11).

Finally, some participants tested in the interest of science or in the interest of the organization facilitating the testing. This reason was labeled '*feeling morally obliged to complete participation in the study*' and is illustrated by the following quotes:

"If everyone starts but, for whatever reason, doesn't finish, that doesn't bring any good to science or [knowledge] dissemination or anyone. So I thought, 'Let me be the person who does do it.'" (T-12).

"I found it nice to know that people are doing this [providing HCV testing]. It gives you the sense that you also need to reciprocate so it won't be one-sided" (T-17).

### Reasons for not testing related to the online testing procedures

From the interviews with participants who did not comply with the test advice, we identified four reasons for not testing related to the online testing service (see lower right section of Figure 1). The first reason reported was that specific features of the laboratories (e.g. opening hours) hindered them from getting tested. This reason was labeled as '*inconvenient testing facilities*' and is illustrated by the following quotes:

"The laboratory which is closest to me is open until, I believe, half past one or half past two [...] and it didn't get to the point that I thought, "Let's go out of bed early to get the test." (non-tester [NT]-15).

"The one that is closest to me - there you can only test by appointment and then I thought, 'Well, I may be in another area someday where there is a lab that doesn't work with appointments and then I'll just walk in to have blood drawn.' That's just more convenient." (NT-6).

Second, the test procedures did not engage participants to commit to taking the test immediately but rather allowed for testing until the end of the year. The testing procedure had therefore an optional, facultative character. This reason was labeled as '*lack of obligatory procedures for testing*' and is illustrated by the following quotes:

"Because I am a diabetic, I have to get blood drawn pretty often, and I thought 'Well, this can wait a little while.' I will certainly do it in time, before December. And that's the deadline you determined" (NT-9).

"I think, for these kind of things, I kind of really need to be ordered to come. It should say, 'Well, on this day at that particular time, you should be there.' Then I would probably free up time for it" (NT-7).

Third, some participants reported '*computer/printing problems*' as a reason for not testing:

"I went to print the form to have the test and my printer broke. It didn't work anymore and I don't have a new printer yet" (NT-11).

"My computer broke down and then I actually didn't end up doing anything with it" (NT-12).

Finally, we found that incorrectly thinking that the online planning tool was a real appointment planner caused uncertainty as to whether the test could still be taken when the planned appointment was skipped. In this situation, we found '*incorrectly interpreting the online planning tool*' to be a reason for not getting tested:

"Well, I skipped the appointment and I didn't know whether I could go another time so I thought, 'Well, then I need to visit my own GP'" (NT-5).

### Reasons for not testing unrelated to the online testing service

We identified eight reasons for not testing that were unrelated to the online testing service (see lower left section of Figure 1). First, despite the results of their online risk assessment, some participants felt they were not at risk or downplayed their reported personal risk for HCV. This reason was labeled as a '*downplaying personal risk*':

"Actually, I naturally assumed that when you receive blood in the hospital, it's fine" (NT-16).

"I got a tattoo in South Africa but, from what I can remember about that tattoo shop, it was hygienic and they always used new needles. At the time, I never had the sense and today I still don't have the sense that I got something, hepatitis C or maybe something else that you can get from unhygienic tattooing." (NT-15)

Second, some participants perceived the likelihood of being HCV-infected as low because they did not have HCV-related symptoms. The '*absence of HCV symptoms as an indication of HCV-negative status*' is illustrated by the following quote:

"Otherwise I would be completely yellow now. In any event, I don't have any symptoms" (NT-10).

Third, some participants mentioned that there was no immediate need to test as they were not suffering from physical distress that disrupted their daily lives. This reason for not testing does not reflect the perception that one is not at risk but rather it reflects a perceived lack of immediate need to test that is rooted in the perception that the potential HCV infection is not a handicap to the participant's daily functioning. The argument '*low perceived urgency for testing due to the absence of physical complaints*' is illustrated by the following quote:

"I don't have any physical complaints now, regardless of whether or not I have it. There's no emergency. [...] And because now I have little, actually, no complaints, it is not on the top of my priority list. It is not something I really have to do." (NT-15).

We also found that some participants perceived that there was little to be gained from diagnosing and treating a long-term persisting infection now instead of later, and therefore postponed testing. The '*low perceived urgency for HCV treatment*' is illustrated by the following:

"It is not something that is life-threatening. It is not like if I don't get treated within a month, I will be dead by next month. You know, because it is such a long time ago" (NT-8).

Furthermore, some participants reported rejecting the test advice in order to prevent emotional worries about being infected. This was labeled as '*avoiding threatening information*' and is illustrated as follows:

"I always think that you shouldn't always take everything to heart because, if you do, you'll feel it yourself too. I always try to be very straight in that. When I have a headache, I don't think, 'Well then, I will probably also have this and that.' [...] It [not seeking testing] is because of that. We shouldn't take everything to heart." (T-11).

In addition, '*fearing the negative consequences of a positive test result*' and the corresponding uncertainty regarding the chain of events following a positive test result was also an impediment to testing:

"Is taking the medication hard? Are you stuck with it for the rest of your life? What is it? What are the risks? I don't have a clue. And imagine that the test result is positive. Then you think, "What am I getting myself into?" (NT-6).

"It is just like [...] pretending it isn't there [...] burying your head in the sand. [...] I just have to, how can I say it, I have to get the courage to take that step. [...] Yes, because imagine that it is not good, you would have never taken that into account." (NT-9).

Some participants mentioned '*discouraging individuals in the social environment*' as a reason for not testing, as illustrated by the following:

"I didn't go and get the test yet because my husband says, 'Well, you don't have to do it.' [...] My children also took a look at the laboratory form and questioned whether it was necessary. [...] Actually, in the beginning, I thought I'd go to the Public Health Service and because other people saw it [the form] and said to me, 'Oh, you don't need to do it,' that's why I haven't done it yet." (NT-13).

Also, '*competing events' *were found to impede testing for HCV, as illustrated by the following quote:

"Unexpectedly, my father had surgery so I am always at the hospital and I haven't been able to do anything for myself. So I haven't tested yet because of these private matters." (NT-8).

Finally, some participants '*thought they had already been tested for HCV elsewhere' *and therefore did not get tested again:

"Yes, I wanted to do the test but my GP had already sent me for extensive blood work because, lately, I haven't been feeling well. Then it turned out that my blood had been tested for almost everything and the results showed that my blood was okay." (NT-3)

### Reasons for intention to test among noncompliant participants

Without explicit solicitation, the majority (11/15) of the participants who did not comply with the test advice expressed the intention to get tested in the future. One reason was the '*ease of testing'*:

"Actually I assume that I'm not infected. That's what plays a role but, because it is that easy, I think, 'Well let's then do it just to be sure'" (NT-6).

Another reason is the '*anonymity of the testing service' *as illustrated by the following quote:

"Where I live - a small village - if you got to the local care unit where blood is drawn, you see all sorts of people you know. If you sit there, then you're either pregnant or you have some scary disease. Well for me, I don't like that, so I'd prefer to go to Amsterdam." (NT-5).

Both of the above-mentioned reasons for intention to test are comparable to reasons for testing mentioned by the compliant participants.

Participants with the intention to test also mentioned reasons related to the benefits of testing. Again, these were identical to those mentioned by the compliant participants. These were '*preventing of liver disease*': "I think everything is okay but there is a chance that I have it. So then maybe it is just better to know and maybe go through nasty treatment for a while so that I don't have complaints later on." (NT-15); '*curability of HCV'*: "Well if you read that if you're infected, it is possible to get treated, then I think, 'Well, maybe I should find that out'" (NT-6); '*seeking reassurance'*: "Yes, I intend to test just to know for sure that it's not there" (NT-13); and '*preventing the further transmission of HVC*': "Yes, well, I have four children so [...] I have another responsibility too. If I were to get sick then I could also infect my children" (NT-5).

## Discussion

The purpose of this study was to gain a better understanding of, firstly, why some people who receive online advice to test for HCV comply with that advice while others do not and, secondly, the role of the online testing procedures in compliance and noncompliance with testing advice. Here, we discuss our findings in relation to existing theory. We suggest methods and strategies to improve not only our online HCV screening project but also other comparable projects that use online tools or aim to encourage individuals to test for HCV.

We found that the autonomous nature of the testing procedure (i.e. with this procedure, it is possible to obtain information independently and to get the test without having to discuss it with a GP) motivated individuals to go for testing. From the theoretical perspective of the theory of planned behavior, the autonomous nature of the service increases perceived behavioral control over the testing procedure as it removes constraining conditions (e.g. having to discuss testing with a GP). The autonomous nature of the service clearly illustrates the added value of internet-based screening projects complementary to existing prevention and screening options. In future screening projects, the autonomous nature of the testing procedure should to serve as the strongest selling-point in communication about the service.

Furthermore, we found that getting an HCV test was also motivated by the fact that knowing one's HCV status can provide reassurance for those who test negative, and can prevent liver disease and inhibit the further transmission of HCV through the initiation of treatment and precautionary measures for those who test positive. Also, knowing that HCV is curable promotes testing. From the perspective of the health belief model, these reasons reflect the expected benefits of testing that can be obtained from either a positive test result followed by treatment and preventive measures or a negative test result (reassurance). We suggest that screening projects communicate not only the physical gains of testing positive but also the emotional benefits that testing negative can potentially offer to especially individuals in low prevalence populations.

As expected, high perceived severity of HCV and high perceived vulnerability to HCV motivated individuals to seek testing. High perceived severity was based on experiences with liver disease in the social environment, where seeing significant others suffer from liver disease increased the will to prevent the disease. High perceived vulnerability was mainly based on the personal test advice that was tailored to the individual's risk profile. The positive effect of tailored health information on screening uptake has been demonstrated previously. For example, Skinner et al. found that among groups with low adherence to breast cancer screening (African American and low-income women), a mammography recommendation letter that was tailored to women's specific health belief model perceptions resulted in a higher mammography adherence at follow-up compared to those who received a nontailored version of the letter [24]. Both perceived vulnerability and severity fit the health belief model, in which especially high perceived vulnerability is an important predictor of performing a desired health behavior [17]. Among the participants in our study, however, sometimes strong feelings of vulnerability were based on previously experiences or events that do not carry any risk for acquiring HCV or on physical complaints unrelated to HCV. For these individuals, the project's threatening information likely created excessive worry that, in turn, motivated testing. Although it was the project's aim to motivate individuals at risk for HCV to seek testing, this finding indicated that presenting threatening information may also motivate the 'worried well' to seek testing. We suggest that HCV screening campaigns increase the perceived relevance of testing for those at risk while also seeking to mitigate the worried well response. This could be done by presenting information about potential personal risk for HCV together with information about the issues that might cause individuals to needlessly worry about HCV (e.g. risks related to other less severe infections).

Furthermore, we found that some individuals got tested to gain a sense of accomplishment and a sense of personal gratification from getting the test that could be interpreted as an anticipated positive emotional reaction. Others claimed to have a moral obligation to complete their participation in the study, which appears to be a form of altruism. Other studies have shown that altruism can indeed motivate participation in research and other projects [e.g. [25]]. Screening projects could use the argument of anticipated gratification in persuasive communication that seeks to enhance compliance with test procedures (e.g. in a reminder message).

Reminder messages and the online planning tool were indicated by some users as facilitative of testing. Reminder messages can be described best as cues to action. They help individuals to recall their initial motivation and rationale to test. The effect of reminders has been demonstrated previously, for example by Sequist et al [26], who showed that colorectal cancer screening rates were higher for patients who received mailings compared with those who did not, and DeFrank et al [27], who showed that reminders were effective in promoting repeat mammography adherence. In our project, we used relatively simple reminder messages that simply stated that individuals should seek testing. The impact of these reminders could likely be improved by including messages that play into the established reasons for testing (e.g. the benefits of testing or the anticipated gratification of finishing the testing procedure).

The online planning tool that was offered to individuals after their online risk assessment supported individuals to set their testing goal and to plan each step toward that goal. This module assisted in closing the gap between intention and behavior and its effect has previously been demonstrated [28]. However, in our study, some individuals did not go for testing because they mistook the online planning tool to be a real appointment planner and, once the planned appointment was missed, they felt uncomfortable making a new appointment. Alternatively, they thought that they could only test at their initially chosen location and time. We suggest that future online planning tools include a clearer explanation of its self-regulating nature in order to prevent the incorrect perception that the planning tool is a real appointment planner. It would also be advantageous to inform those who miss their planned test date that it is possible to get the test at another moment in time. In addition, online planning tools could incorporate email or SMS reminder messages that, for example, a day prior to the planned appointment, send a reminder message in which the personal goal (i.e. getting tested at a particular date, location, and time) is reiterated.

With respect to the reported reasons for not using the online HCV testing service, we found that the lack of obligatory procedures for testing and inconvenient testing facilities impeded testing. Although these aspects reflect the autonomous nature of the testing service, which did motivate most individuals to seek testing, some found these very same features to be barriers to their use of the service. Individuals had to plan the HCV testing appointment themselves and the online service did not incorporate any procedures that produce high commitment for testing (e.g. scheduling real appointments). Moreover, the online testing procedure unintentionally offered a cue for procrastination as it explicitly indicated a lenient deadline for testing (i.e. a maximum of 12 months). We suggest that online screening projects in the future provide individuals with a clearly defined and relatively tight deadline for testing. We recommend a period of one month as the evaluation study of the HCV internet project (data not published) showed that most individuals were tested within two weeks.

Furthermore, we found that low perceived urgency for testing and treatment impeded testing for HCV. From the perspective of the health belief model, this reason reflects low perceived severity of HCV infection which can lead to procrastination in testing. Although it is true that, in most cases, HCV is not an acute life-threatening disease that demands immediate treatment, individuals cannot precisely know the degree to which their (potential) infection has progressed. We thus suggest that future HCV screening projects emphasize this and seek to deter the notion that HCV treatment can be easily postponed. This could be done by outlining both the negative consequences of postponing an HCV test and the benefits of immediate testing and subsequent treatment.

Additional reasons for noncompliance with HCV test advice were that no symptoms were present and that the risk was downplayed. These reflect low perceived vulnerability of HCV infection. The absence of symptoms as an indication that no infection has occurred might be based on a false belief that all HCV infections are accompanied by physical symptoms. Interestingly, the information provided by the testing service did indicate that the majority of HCV infections are asymptomatic. The fact that individuals mentioned these reasons for not testing despite the provision of appropriate information and personalized advice to seek testing may reflect unrealistic optimism, which is an optimistic bias regarding personal vulnerability to a health threat ["It won't happen to me"; [29]]. With this in mind, we suggest the use of scenario-based risk information that addresses doubts about personal risks and the consequences of downplaying of risk. For example, future efforts to promote compliance with test advice could use the story of a HCV-infected peer who was diagnosed late because he did not experience any symptoms and thought that his chance of having acquired HCV was small.

Although low perceived severity and vulnerability (representing low perceived threat) can lead to procrastination or noncompliance with testing advice, we should be careful with respect to increasing perceived threat as we found that some individuals showed testing avoidance because of a high perceived threat. For these individuals, avoidance of threatening information and fear of the consequences of a possible positive test result impeded testing. As such, the advice to seek testing may have resulted in a fear control reaction as described by the extended parallel process model. According to this model, high perceived threat in combination with low perceived efficacy for testing can lead to maladaptive responses such as denial of the message and message avoidance. Therefore, screening projects should not only seek to address personal risk and increase the perceived health threat of HCV; they should also endeavor to increase individuals' perceived response and self efficacy for managing a possible infection. In our project, we informed individuals about the blood testing procedure but we did not specifically mention the face-to-face post-test counseling session with a trained professional that always follows a positive test result. We, therefore, recommend that screening projects provide more detail on the procedures that follow a positive test result. Online screening projects could also incorporate opportunities for an immediate online post-test counseling session (e.g. via webcam) in addition to face-to-face counseling. Furthermore, they could include an online module that teaches individuals the necessary skills to overcome their fear of a positive test result by, for example, arranging support from family members.

Some participants indicated that individuals in their direct social environment discouraged them from testing for HCV. According to the theory of planned behavior, a strong negative subjective norm (i.e. others' beliefs regarding testing plus the motivation to comply with the beliefs of others) can influence testing behavior. In order to overcome social pressure not to test, screening projects can offer skill-building tools that help individuals to negotiate or withstand discouragement from their environment. Scenarios that offer counterarguments against a discouraging partner or demonstrate how to surpass social pressure and maintain the original testing intention would be beneficial in this regard.

Some participants reported a malfunctioning computer or printer as a barrier to testing. In our project, individuals could have their laboratory form emailed to them or they could download it onto their computer but we did not actively offer individuals a solution to printing problems. This technical problem could be overcome in future screening projects by offering to send the laboratory form by post or by having the laboratory forms emailed to mobile phones or to the laboratories directly.

Finally, competing events impeded testing. Most of the reported competing events were very serious (e.g. hospitalization of a family member). Consequently, the low prioritization of HCV testing by these participants seems reasonable. Given this finding, we suggest that future HCV screening projects incorporate a multiple reminder system in which individuals are reminded of testing not only a couple of days after their risk assessment, as in our project, but also a couple of weeks later.

Although our study focused on reasons for compliance and noncompliance with advice to test for HCV, some noncompliant participants mentioned that they still intended to test. These participants provided similar reasons for testing as the advice-compliant participants. This suggests that the reasons for testing and the reasons for not testing may have played a role in both testers' and non-testers' decision-making. It would be interesting to further investigate what discriminates individuals who eventually test from those who do not. It could be that testers encountered the impeding factors to a lower extent or that they overcame these factors better than non-testers. Quantitative studies in the future could provide further insight regarding the presence and strength of the various reasons among both groups and their relation to testing.

To our knowledge, this is the first study to explore reasons for compliance and noncompliance with an HCV test advice in the general population. Previous studies have been conducted among drug users [30-32]. In these studies, some of the reasons for testing or not testing for HCV were similar to those identified by our study. For example, these studies also found that a motivating factor is that the test enables avoidance of the GP and an impeding factor is low perceived risk of being infected. However, the drug users in these studies rarely mentioned reasons for testing related to health benefits. They also mentioned many dissimilar reasons for noncompliance such as fear of needles, perceived lack of confidentiality regarding test results, and fear of discrimination and stigmatization. This seems to suggest that HCV testing projects targeting active drug users should have a different focus (e.g. focus on issues relevant to drug users' lifestyles and competing problems) than HCV testing projects targeting the general population.

Our study has a number of limitations. First, the participants were individuals who had responded to a HCV campaign, completed the online risk assessment questionnaire, and left their email address. Individuals who did not respond to the campaign or who left the website before completing the risk assessment questionnaire were not invited to participate. This could generate a selection bias whereby our study sample includes relatively more individuals who were informed and committed to the service. Also, women and individuals of Dutch origin dominated the study sample and not all risk groups for HCV were represented (e.g. individuals born to an HCV-positive mother). Future research should focus on the reasons for (non-)participation of these groups.

## Conclusions

This study has shown that our online screening campaign motivated individuals to test because the testing service is autonomous, because tailored risk information is provided, because a reminder message service is in place, and because there is an online planning tool. Furthermore, our study elicited a number of feasible intervention targets to improve the uptake of HCV testing in general. We suggest that HCV screening projects include a deadline for testing and anticipate the responses of individuals with low perceived risk for HCV by, for example, raising awareness of personal risk and outlining the consequences of not testing. Also, projects could communicate the emotional benefits of testing negative in addition to the physical gains of testing positive. Furthermore, projects could provide additional insight regarding the procedures that follow a positive test result. We propose that organizing an effective low threshold HCV testing procedure for the general population could not have been successful without the internet. Given its tailoring capabilities, flexibility, and the relatively low costs, the internet is a promising tool not only for arranging HCV testing but also for motivating individuals to get tested by providing them with advice based on a personal risk profile. In addition, we believe that the anonymous character of the internet and subsequent testing procedures are especially helpful for addressing stigmatized diseases like HCV.

## Competing interests

The authors declare that they have no competing interests.

## Authors' contributions

FRZ coordinated the study, carried out the interviews, participated in the data analyses and interpretation, and drafted the manuscript. TRLJH participated in the data analyses and interpretation, and contributed to the drafting of the manuscript. ATU carried out the interviews and participated in the data analyses, and contributed to the drafting of the manuscript. MP contributed to the study design, the interpretation of the results, and drafting of the manuscript. GK contributed to the interpretation of the results and drafting of the manuscript. UD conceived and supervised the study and analyses, and led the writing. All authors read and approved the final manuscript.

## Pre-publication history

The pre-publication history for this paper can be accessed here:

http://www.biomedcentral.com/1471-2458/11/293/prepub
